# Comparative study of MR mTI-ASL and DSC-PWI in evaluating cerebral hemodynamics of patients with Moyamoya disease

**DOI:** 10.1097/MD.0000000000012768

**Published:** 2018-10-12

**Authors:** Jinge Zhang, Chunchao Xia, Yi Liu, Weiqiang Qian, Wanlin Peng, Keling Liu, Lei Li, Fei Zhao, Zhenlin Li

**Affiliations:** a Department of Radiology; b Department of Neurosurgery, Sichuan University West China Hospital, Chengdu City, China.

**Keywords:** ASL, DSC-PWI, Moyamoya disease, perfusion imaging

## Abstract

The aim of this study was to explore the correlation between multi-inversion time arterial spin labeling (mTI-ASL) and dynamic susceptibility contrast-enhanced perfusion weighted imaging (DSC-PWI) in assessment of hemodynamics of patients with Moyamoya disease (MMD).

In this study, 24 MMD patients and 21 healthy subjects were enrolled between June 2017 and December 2017. The images of mTI-ASL and DSC-PWI in the week before revascularization surgery were retrospectively analyzed. The parameters of cerebral blood flow (CBF), time to peak (TTP), and bolus arrival time (BAT) were measured in regions of interest (ROIs) of lateral middle cerebral artery (MCA) territories, basal ganglia (BG), and cerebellum, and relative perfusion parameters (rCBF-ASL, rBAT-ASL, rCBF-DSC, and rTTP-DSC) were calculated by dividing by cerebellum value. One-way analysis of variance and Student–Newman–Keuls tests were performed to compare rCBF-ASL and rCBF-DSC in the MMD group and the control group. Unpaired *t* test was used to compare rBAT-ASL and rTTP-DSC in the MMD group and the control group. And we assessed the correlation between rCBF-ASL and rCBF-DSC and between rBAT-ASL and rTTP-DSC using Pearson correlation analysis.

All the relative parameters were significantly different between the MMD group and the control group (all *P*<.05). Meanwhile, there was significant difference between rCBF-ASL and rCBF-DSC (*P*<.05), and there was strong correlation between rCBF-ASL and rCBF-DSC (r = 0.839, *P*<.001), and moderate correlation between rBAT-ASL and rTTP-DSC (r = 0.519, *P*<.001).

Both mTI-ASL and DSC-PWI could be used to assess perfusion state in MMD patients before revascularization surgery effectively. As a noninvasive imaging technique, mTI-ASL could provide perfusion parameters without contrast medium injection, and the results were quite correlative with DSC-PWI.

## 1 Introduction

Moyamoya disease (MMD) is a rare cerebrovascular occlusive disease that is characterized by progressive stenosis of distal of the internal carotid arteries (ICA) around the circle of Willis,^[1]^ which leads to the formation of abnormal collateral vascular networks that look like a “puff of smoke” at the skull base.^[2]^ MMD was first identified by Suzuki and Takaku^[3]^ in 1969 and lots of clinical studies have expanded our knowledge of MMD. Many latest researches have indicated that the vascular involvement (stenosis and occlusion) of ICA, anterior cerebral artery (ACA), and middle cerebral artery (MCA) will change the cerebral hemodynamics in MMD patients.^[4,5]^ And varieties of perfusion imaging techniques provide a way to evaluate condition of MMD patients. According to the guidelines for the diagnosis and treatment of MMD, only patients with obvious symptoms and evidence-based perfusion defects need to receive surgical treatment.^[6]^ Therefore, it is critical to assess MMD patients’ cerebral perfusion before clinical treatment.^[7]^

To date, positron emission tomography (PET) and single-photon emission computed tomography (SPECT) are the gold-standard examinations for cerebral perfusion assessment.^[6]^ Meanwhile computed tomography perfusion (CTP) imaging and magnetic resonance (MR) dynamic susceptibility contrast-enhanced perfusion weighted imaging (DSC-PWI) provide more convenient and less expensive methods, and have been widely used.^[8,9]^ In DSC-PWI, the paramagnetic intravenous contrast agent remains in intravascular space and causes field inhomogeneity. This results in increased magnetic susceptibility heterogeneity and reduction of signal intensity on T2 weighted images during the first pass of agent through the brain vasculature.^[10]^ According to the curves of signal change, parameters of cerebral blood flow (CBF), cerebral blood volume (CBV), time to peak (TTP), and mean transmit time (MTT) can be calculated. Because it involves the use of nonionizing radiation, DSC-PWI is most commonly used to assess cerebral perfusion.^[11]^ Numerous studies have proved that the results of DSC-PWI are well correlated with those of SPECT in evaluating cerebral hemodynamics.^[12,13]^

Nevertheless, DSC-PWI still has some disadvantages, including the need to administer a contrast medium and the risk of high-pressure bolus injection. Different from DSC-PWI, arterial spin labeling (ASL) is a noninvasive MR perfusion imaging technique.^[14]^ ASL uses the arterial blood labeled by radio-frequency pulses as an endogenous contrast medium, and can completely avoid the risk of side effects and high-pressure injection.^[15]^ According to different labeling methods, ASL techniques are divided into several kinds, such as continuous-ASL (CASL), pulsed-ASL, and pseudo-continuous-ASL .^[16]^ Several studies have used ASL imaging technique to assess CBF changes in MMD patients.^[17–19]^ However, most of these studies adopted a single inversion time ASL (sTI-ASL) sequence, which has the disadvantage of measuring only 1 semiquantitative parameter of CBF. Multi-inversion time ASL (mTI-ASL, belong to pulsed-ASL) is an advanced ASL technique that can not only detect both CBF and bolus arrival time (BAT, which represents the time it takes for the labeled blood from tagging plane to the imaging voxel) but also make more accurate quantification of CBF by including BAT measurement.^[20]^ The purpose of this study was to compare the results obtained by mTI-ASL and DSC-PWI in MMD patients.

## 2 Methods

### 2.1 Patients

Twenty-four MMD patients (13 males and 11 females; age range, 19–62 years; mean age, 37.2 ± 15.2 years) diagnosed by digital subtraction angiography (DSA), computed tomography angiography (CTA), or time of flight-magnetic resonance angiography (TOF-MRA) between June 2017 and December 2017 were selected as the MMD group. All of these MMD patients had obvious symptoms (such as hemiplegic paralysis or numbness, aphasia, headache, and dizziness) and undergone surgery (combination of bypass surgery and encephalo-myo-synangiosis, 10 on the left and 14 on the right) in West China Hospital of Sichuan University. The patients who had old cerebral infraction or hemorrhage (>10 days) and who had disease in posterior cerebral circulation were excluded. The images of MR mTI-ASL and DSC-PWI made in the week before operation were retrospectively analyzed.

For a comparative study, 21 healthy subjects (11 males and 10 females; age range, 24–65 years; mean age, 39.6 ± 12.9 years) who did not have disease in cerebral vessels and any noticeable neurologic symptoms (proved to be healthy by other imaging examinations and clinical observation) were enrolled as the control group.

All of the MR images were retrospectively analyzed and written informed consent was obtained from all patients or their next of kin before the contrast-enhanced MR examination.

### 2.2 Imaging protocol and postprocessing procedure

MR mTI-ASL and DSC-PWI scans were carried out as part of the routine clinical brain MR perfusion examinations performed on a 3T MR scanner (Skyra, Siemens Medical Systems, Erlangen, Germany) with a 20-channel head-neck coil. These 2 kinds of imaging methods were done at the same examination, and mTI-ASL sequence were scanned before DSC-PWI. The scanning parameters are presented in Table 1.

**Table 1:**
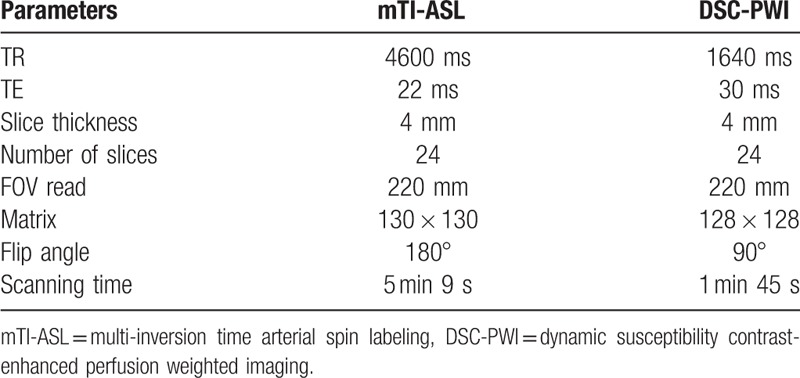
Scanning parameters of mTI-ASL and DSC-PWI.

### 2.3 mTI-ASL imaging

In this study, we applied a prototype sequence of three-dimensional (3D) GRASE mTI-ASL to simultaneously measure CBF and BAT of the MMD group and the control group with the following parameters: TR: 4600 ms; TE: 22 ms; slice thickness: 4 mm; number of slices: 24; FOV read: 220 mm; matrix: 130 × 130; flip angle: 180°; PAT mode: GRAPPA; bolus duration: 700 ms; 1 average per TI, 16 TIs from 480 to 4080 ms (480, 720, 960, 1200, 1440, 1680, 1920, 2160, 2400, 2640, 2880, 3120, 3360, 3600, 3840, 4080), and total scanning time was 5 minutes 9 seconds including an M0 scan. The CBF, BAT, and residual error maps were automatically calculated and drawn on the scanning workstation after mTI-ASL scanning.

### 2.4 DSC-PWI imaging

DSC-PWI images were acquired with an echo-planar imaging sequence using the following parameters: TR: 1640 ms; TE: 30 ms; slice thickness: 4 mm; number of slices: 24; FOV read: 220 mm; matrix: 128 × 128; flip angle: 90°; number of acquisitions: 60; scanning time: 1 minute 45 seconds; and a gadolinium contrast medium (Gd-DTPA, Magnevist, Beijing BeiLu Pharmaceutical Co, Ltd, Beijing, China) was intravenously injected using a high-pressure injector (Medrad Spectris Solaris EP MR injection system, Bayer HealthCare, Whippany, NJ) at the third acquisition (0.2 mL/kg, 4.5–5 mL/s, and followed immediately by a 30 mL physiological saline flush). All original data and images were sent to a Siemens Syngo MMWP VE40A postprocessing workstation and were analyzed with the MR Perfusion software. Maps of CBF, CBV, TTP, and MTT were generated using the local AIF mode.

### 2.5 Calculation of relative perfusion parameters

**Figure 1:**
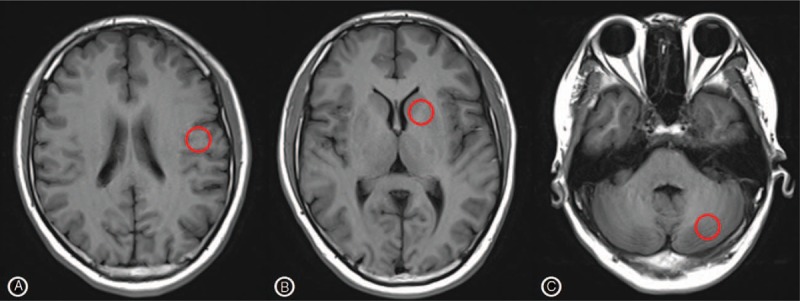
ROI drawing (A) lateral MCA territories, (B) BG, (C) cerebellum. ROI = region of interest, MCA = middle cerebral artery.

DSC-PWI and mTI-ASL images were analyzed on 3 tomographic planes of slice levels passing through the lateral ventricle, middle basal ganglia (BG), and cerebellum hemisphere. Hemodynamic parameters (CBF-ASL, BAT-ASL, CBF-DSC, and TTP-DSC) were measured on each tomographic plane by manually placing the region of interest (ROI) for lateral MCA territories, BG, and cerebellum.^[21]^ The diameter of the ROI was about 10 mm, and we carefully avoided great cerebral vessels and the area with ischemic or hemorrhage lesions when we put ROI (Fig. 1).

The relative parameters (rCBF-ASL, rBAT-ASL, rCBF-DSC, and rTTP-DSC) of each ROI for lateral MCA territories and BG were calculated by dividing by cerebellum value of same side (for example, rCBF-ASL_right__BG_ = CBF-ASL_right__BG_/CBF-ASL_right__cerebellum_). In the MMD group, the operation side (10 on the left and 14 on the right) of each patient was selected to be analyzed, while both sides of each patient were analyzed in the control group.

### 2.6 Statistical analysis

Data were expressed as mean ± SD. The difference between rCBF-ASL of the MMD group, rCBF-ASL of the control group, rCBF-DSC of the MMD group and rCBF-DSC of the control group were examined by one-way analysis of variance and Student–Newman–Keuls test in ROIs for lateral MCA territories and BG, respectively. And unpaired *t* test was used to compare the rBAT-ASL and rTTP-DSC between the MMD group and the control group, respectively. The relationships between rCBF-ASL and rCBF-DSC and between rBAT-ASL and rTTP-DSC in all ROIs were quantified by Pearson correlation analysis. Statistical significance was set at the *P*<.05 level. All of the statistical analyses were performed using the SPSS 19.0 software (SPSS Inc, Chicago, IL).

## 3 Results

### 3.1 Baseline characteristics

The baseline characteristics were presented in Table 2. In this study, in total 24 MMD patients and 21 healthy subjects were enrolled. In MMD group, all the 24 patients had obvious symptoms such as hemiplegic paralysis or numbness (5 patients), aphasia (4 patients), headache (14 patients), and dizziness (19 patients). Six patients had cerebral ischemia and 4 patients had hemorrhage. Vascular involvement was unilateral in 9 (4 left, 5 right) patients and bilateral in 15 patients. There was no significant difference between the age of the MMD group and the control group (t = 0.578, *P*>.05). And paired *t* test showed that there was no significant difference between perfusion parameters of bilateral hemisphere in control group (all *P*>.05, presented in Table 3).

**Table 2:**
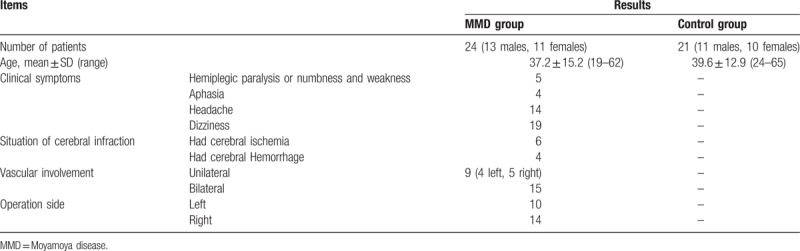
Baseline characteristics.

**Table 3:**
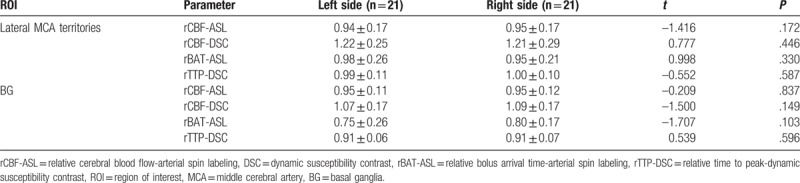
Paired *t* test of bilateral hemisphere of the control group.

### 3.2 Comparison of rCBF-ASL and rCBF-DSC of MMD group and control group

**Figure 2:**
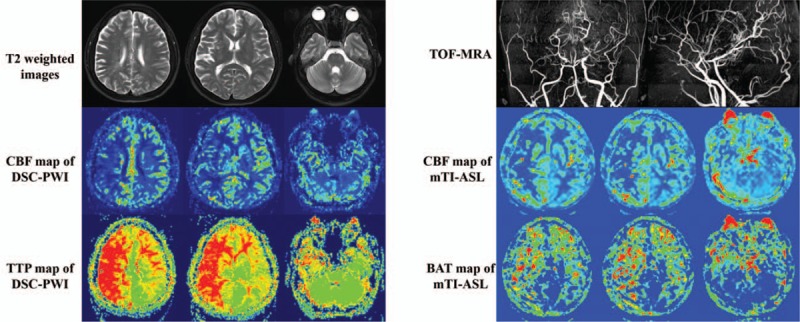
A 37-year-old female patient was hospitalized for TIA and cerebral hemorrhage and was diagnosed with MMD by TOF-MRA. The results of DSC-PWI showed prolonged TTP and decreasing CBF in right MCA territories. In mTI-ASL images, it could be observed that the area of prolonged BAT matched the TTP-DSC map closely, while the area of decreasing CBF was also similar to DSC-PWI images. MMD = Moyamoya disease, TOF-MRA = time of flight-magnetic resonance angiography, DSC-PWI = dynamic susceptibility contrast-enhanced perfusion weighted imaging, TTP = time to peak, CBF = cerebral blood flow, MCA = middle cerebral artery, mTI-ASL = multi-inversion time arterial spin labeling, BAT = bolus arrival time.

Among the 24 MMD patients, varying degrees of hypoperfusion areas were observed on the map of CBF-ASL, BAT-ASL, CBF-DSC, and TTP-DSC (Fig. 2).

**Figure 3:**
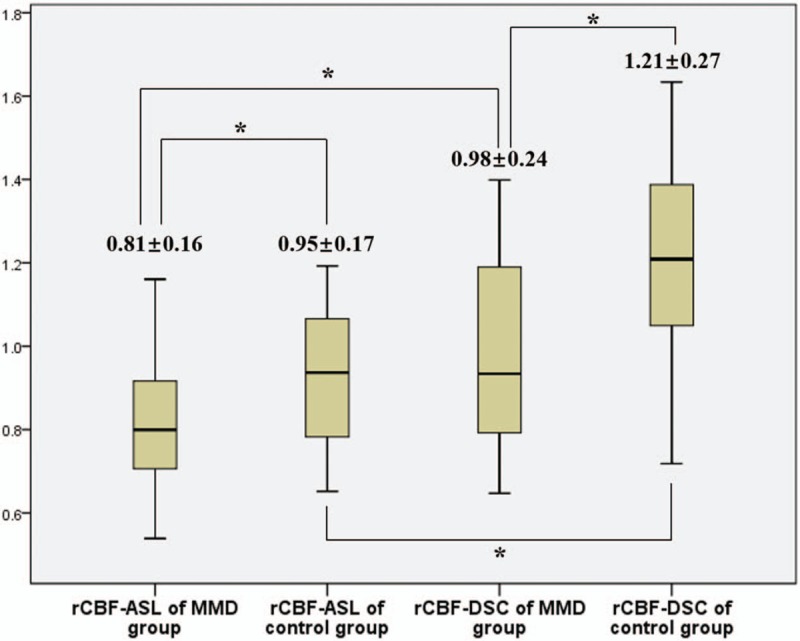
rCBF in ROIs of lateral MCA territories (^∗^significant difference between 2 groups, *P*<.05). rCBF = relative cerebral blood flow, ROI = region of interest, MCA = middle cerebral artery.

In ROIs of lateral MCA territories, the average values of rCBF-ASL of the MMD group and the control group were 0.81 ± 0.16 and 0.95 ± 0.17, respectively, while rCBF-DSC of the MMD group and the control group were 0.98 ± 0.24 and 1.21 ± 0.27. One-way analysis of variance revealed that there was significant difference between the 4 groups of rCBF values (F = 20.37, *P*<.001). And multiple pairwise comparison of Student–Newman–Keuls test revealed that both rCBF-ASL and rCBF-DSC were significantly different between the MMD group and the control group, while the rCBF-ASL was significantly different from rCBF-DSC in both the MMD group and the control group (all *P*<.05, Fig. 3).

**Figure 4:**
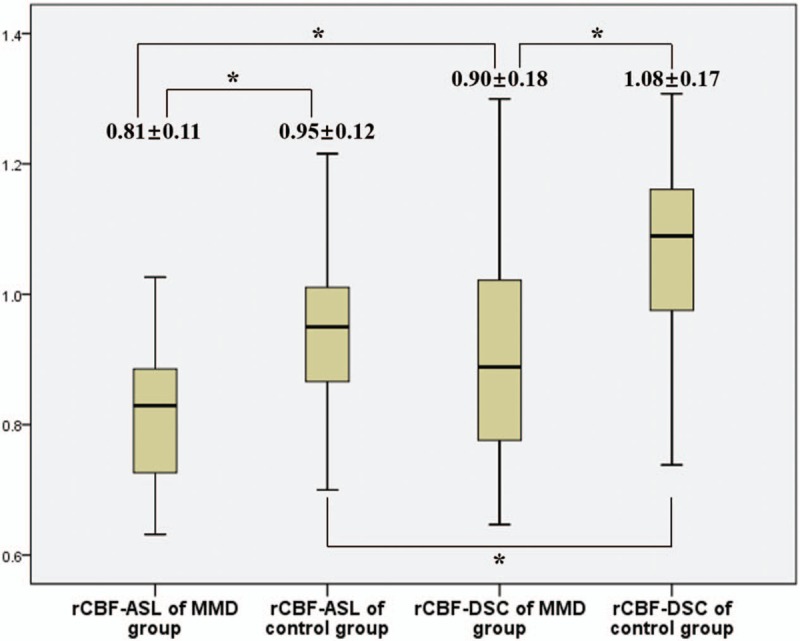
rCBF in ROIs of BG (^∗^significant difference between 2 groups, *P*<.05). rCBF = relative cerebral blood flow, ROI = region of interest, BG = basal ganglia.

In ROIs of BG, the average values of rCBF-ASL of the MMD group and the control group were 0.81 ± 0.11 and 0.95 ± 0.12, respectively, while rCBF-DSC of the MMD group and the control group were 0.90 ± 0.18 and 1.08 ± 0.17. The results of comparison were similar to that of lateral MCA territories and shown in Fig. 4.

### 3.3 Comparison of rBAT-ASL and rTTP-DSC between the MMD group and the control group

The results of the unpaired *t* test are shown in Table 4. In ROIs of lateral MCA territories, rBAT-ASL of the MMD group (1.23 ± 0.39) and the control group (0.97 ± 0.24) were significantly different (*P*< .01), rTTP-DSC of the MMD group (1.08 ± 0.10) and the control group (0.99 ± 0.10) were also significantly different (*P*<.01). In ROIs of BG, rBAT-ASL of the MMD group (0.96 ± 0.28) and the control group (0.77 ± 0.22) were significantly different (*P*<.01), rTTP-DSC of the MMD group (0.98 ± 0.06) and the control group (0.91 ± 0.06) were also significantly different (*P*<.01).

**Table 4:**
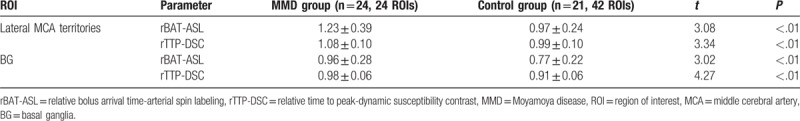
Unpaired *t* test of rBAT-ASL and rTTP-DSC between the MMD group and the control group.

To sum up, both rBAT-ASL and rTTP-DSC were significantly different between the MMD group and the control group.

### 3.4 Relationship between rCBF-ASL and rCBF-DSC and between rBAT-ASL and rTTP-DSC in all ROIs

**Figure 5:**
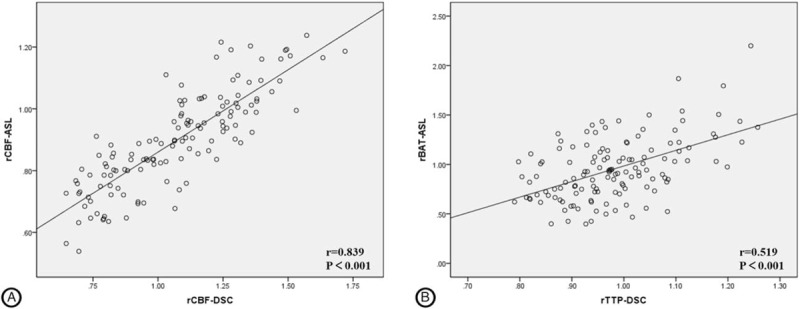
A, Correlation between rCBF-ASL and rCBF-DSC in all the 90 ROIs of lateral MCA territories and BG. B, Correlation between rBAT-ASL and rTTP-DSC in all the 90 ROIs of lateral MCA territories and BG. rCBF-ASL = relative cerebral blood flow-arterial spin labeling, DSC = dynamic susceptibility contrast, ROI = region of interest, MCA = middle cerebral artery, BG = basal ganglia.

For the whole 132 ROIs (48 ROIs in MMD group and 84 ROIs in control group) of lateral MCA territories and BG in the MMD group and the control group, a strong correlation was observed between rCBF-ASL and rCBF-DSC (r = 0.839, *P*<.001, shown in Fig. 5), While rBAT-ASL had moderate correlation with rTTP-DSC (r = 0.519, *P*<.001, shown in Fig. 5).

## 4 Discussion

There are 3 main findings in our study. First, rCBF-ASL and rBAT-ASL calculated from ASL parameters map were significantly different between the MMD group and the control group, as well as rCBF-DSC and rTTP-DSC. The imaging technique of mTI-ASL could detect hypoperfusion areas of MMD patients and evaluate perfusion state quantitatively by the map of CBF and BAT. Second, although the value of rCBF was normalized by dividing by cerebellum value, calculated rCBF-ASL was significantly lower than rCBF-DSC, both in ROIs of lateral MCA territories and BG. Because only a small portion of these patients underwent PET or SPECT examinations, we could not compare the 2 groups of rCBF with the results of the gold standards. So, it was uncertain whether mTI-ASL or DSC-PWI could obtain a more accurate value of CBF. Noguchi et al^[5]^ compared the results of ASL and SPECT, and also found that ASL tended to underestimate CBF, which is a limitation of the ASL technique. But the reason needs further investigation. Third, there was very strong correlation between rCBF-ASL and rCBF-DSC (r = 0.839, *P*<.001) and moderate correlation between rBAT-ASL and rTTP-DSC (r = 0.519, *P*<.001). BAT and TTP have different meanings in perfusion evaluation although they are both associated with the progression of MMD, and that might be the reason why there was not strong correlation between them. BAT means the time it takes for the blood to move from the labeling plane to the imaging plane. It reflects the delay in blood supply to the tissue because of narrowing of large vessels, whereas TTP reflects the situation of large vessels and small vessels.^[22,23]^

Zhang et al^[19]^ analyzed the results of 3D-ASL and DSC-PWI in 28 ischemic stroke patients and found there were moderate correlations (r values, 0.531–0.611) between rCBF-ASL and rCBF-DSC in different groups of ROIs. Qiao et al^[11]^ compared mTI-ASL and DSC-PWI parameter maps in 41 patients with MMD and found the correlations between rCBF-ASL and rCBF-DSC (r = 0.316, *P*<.001) and between rBAT-ASL and rTTP-DSC (r = 0.357, *P*<.001) were not strong. The difference between the results of our study and theirs might have the following reasons. First, different modalities and imaging techniques may cause the difference in data. Compared with other sTI-ASL imaging techniques, the mTI-ASL sequence could simultaneously measure multiple parameters, not only CBF, but also the time-dependent parameters—BAT. What is more, it also makes more accurate quantification of CBF by including BAT measurement.^[20]^ Second, calculations of rCBF, rBAT, and rTTP were conducted with an unusual method in our study. The relative parameters are usually calculated by dividing the value of drawn ROI by its mirror area in perfusion imaging analysis.^[19]^ Considering that many MMD patients were involved in bilateral ICAs, ACAs, or MCAs, we chose cerebellum supplied by posterior circulation as the standardized reference to calculate relative parameters,^[21,24]^ so that we could get more accurate data to reflect the degree of hypoperfusion in ROI. And in our study, all 45 patients were normal in posterior circulation according to clinical observation and MR examination.

Changes in hemodynamics are closely associated with progression of MMD, so that the assessment of cerebral perfusion state is critical for treatment selection.^[7]^ As mentioned earlier, there are several perfusion imaging techniques for cerebral perfusion evaluation. SPECT and PET are the gold-standards, while CTP and DSC-PWI provide a more convenient method and are developing rapidly. However, although techniques of low-dose CTP scanning are widely used in clinical application nowadays, radiation dose is still the biggest obstacle to popularizing the use of CTP with the public's growing recognition of the importance of radiation protection.^[25]^ Compared with DSC-PWI, ASL-MRI is a noninvasive imaging technique and can avoid these risks. But it is not perfect; the scanning time of ASL is much more than that of DSC-PWI, which leads to more sensitivity to motion artifact. More than that, routine ASL techniques can only get the map of CBF, while DSC-PWI can provide multiple parameters of CBF, CBV, MTT, and TTP. In current perfusion imaging studies about ischemia, the degree of decrease in CBF and prolongation in TTP or MTT could reflect the severity of cerebral ischemia, but the time parameters of TTP and MTT are more sensitive.^[7,26]^ Lee et al^[24]^ indicated that a combination of TTP and CBF could be used to evaluate the ischemia state of MMD patients effectively. The multi-inversion time ASL (mTI-ASL) used in our study can measure CBF and BAT simultaneously, which allows for more comprehensive and accurate assessment of the cerebral hemodynamic state in MMD patients.

The main limitation of our study was that mTI-ASL and DSC-PWI were not compared with gold-standards, namely, SPECT or PET, because of a lack of data. Meanwhile, the sample size was small. Further research is needed to explore the value of mTI-ASL in perfusion evaluation of MMD patients.

In conclusion, both mTI-ASL and DSC-PWI can detect the area of hypoperfusion of MMD patients and evaluate the cerebral hemodynamic state quantitatively, and the perfusion parameters of them are well correlated. As a noninvasive and multiparameter perfusion imaging technique, mTI-ASL is potentially valuable in preoperative assessment and treatment decision-making of MMD patients. Although there are only a few literatures about application of this emerging technique, and all of them are researches about cerebral hemodynamics,^[11,20,22]^ we can still expect that mTI-ASL will be used in more body parts and more diseases.

## Author contributions

**Conceptualization:** Chunchao Xia.

**Data curation:** Weiqiang Qian, Fei Zhao.

**Formal analysis:** Yi Liu.

**Investigation:** Weiqiang Qian.

**Methodology:** Yi Liu.

**Project administration:** Zhenlin Li.

**Software:** Jinge Zhang, Wanlin Peng, Keling Liu, Lei Li.

**Writing – original draft:** Jinge Zhang.

**Writing – review & editing:** Zhenlin Li.

Jinge Zhang orcid: 0000-0001-5045-6530.
