# Comparative study of MR mTI-ASL and DSC-PWI in evaluating cerebral hemodynamics of patients with Moyamoya disease: Erratum

**DOI:** 10.1097/MD.0000000000013497

**Published:** 2018-11-21

In the article, “Comparative study of MR mTI-ASL and DSC-PWI in evaluating cerebral hemodynamics of patients with Moyamoya disease”,^[1]^ which appears in Volume 97, Issue 41 of *Medicine*, the caption for Figure 5 is incorrect and should be “A, Correlation between rCBF-ASL and rCBF-DSC in all the 132 ROIs of lateral MCA territories and BG. B, Correlation between rBAT-ASL and rTTP-DSC in all the 132 ROIs of lateral MCA territories and BG. rCBF-ASL = relative cerebral blood flow-arterial spin labeling, DSC = dynamic susceptibility contrast, ROI = region of interest, MCA = middle cerebral artery, BG = basal ganglia.”
